# Novel Insights into PGM2L1 as a Prognostic Biomarker in Cholangiocarcinoma: Implications for Metabolic Reprogramming and Tumor Microenvironment Modulation

**DOI:** 10.7150/ijms.106566

**Published:** 2025-02-11

**Authors:** Guo-Wei Wu, Yi-Hsien Hsieh, Yi-Chung Chien, Li-Yuan Bai, Yung-Luen Yu

**Affiliations:** 1 Graduate Institute of Biomedical Sciences, China Medical University, Taichung 406040, Taiwan.; 2 Institute of Translational Medicine and New Drug Development, China Medical University, Taichung 406040, Taiwan.; 3 Center for Molecular Medicine, China Medical University Hospital, Taichung 404327, Taiwan.; 4 Institute of Medicine, Chung Shan Medical University, Taichung 40201, Taiwan.; 5 Department of Medical Research, Chung Shan Medical University Hospital, Taichung 40201, Taiwan.; 6 Division of Hematology and Oncology, China Medical University Hospital, Taichung, 404327, Taiwan.; 7 Department of Medical Laboratory Science and Biotechnology, Asia University, Taichung 413305, Taiwan.

**Keywords:** cholangiocarcinoma, PGM2L1, tumor-infiltrating immune cells, glycolytic metabolism

## Abstract

Cholangiocarcinoma (CCA) is a highly lethal malignancy and the most common adenocarcinoma of the hepatobiliary system. PGM2L1 belongs to the α-D-phosphohexomutase superfamily and functions as a glucose 1,6-bisphosphate (G16BP) synthase. There is growing evidence to provide an association its function to cancer metabolism and progression. However, the molecular mechanisms of PGM2L1 in CCA development remain lacking in evidence. In this study, we found that CCA patients with high PGM2L1 expression had the poorest prognosis. We identified two methylation sites (cg15214137 and cg03699633) within the PGM2L1 gene and their prognostic relevance. We further investigated the relationship between PGM2L1 expression and tumor-infiltrating immune cells, with a particular focus on neutrophils in CCA. Functional enrichment analyses further revealed that high PGM2L1 expression was associated with the Wnt signaling pathway, glycolytic metabolism, and the recruitment of neutrophils. Collectively, these findings suggest that PGM2L1 may serve as an independent prognostic biomarker and is closely linked to tumor immune infiltration and metabolic reprogramming in CCA.

## 1. Introduction

Cholangiocarcinoma (CCA) is a malignant adenocarcinoma originating from the epithelial cells lining the biliary tree. It is highly lethal and heterogeneous within the hepatobiliary system. Cholangiocarcinoma is classified into three subtypes based on its anatomical origin: intrahepatic cholangiocarcinoma (iCCA) when located above the second-order bile ducts within the liver parenchyma, perihilar cholangiocarcinoma (pCCA) occurring at the hepatic hilum, and distal cholangiocarcinoma (dCCA) located in the common bile duct below the insertion of the cystic duct. Among them, many databases classify both pCCA and dCCA as extrahepatic CCA 1, 2. In addition, each subtype exhibits unique genetic abnormalities, clinical manifestations, and treatment strategies. Treatment options for CCA include surgery, chemotherapy, radiation therapy, targeted therapy, immunotherapy, and transarterial chemoembolization (TACE), among others. Currently, the first-line treatment modalities employed are early surgical resection or transarterial chemoembolization (TACE). However, most patients afflicted with this condition do not present at an early stage 3, 4. Therefore, for advanced-stage disease, the treatment approach involves utilizing second-line chemotherapy drugs, such as the combination of gemcitabine and cisplatin. As for the third-line treatment, targeted therapy options like Pemigatinib which is the FGFR2 inhibitor or Bevacizumab (Avastin) which is the vascular endothelial growth factor (VEGF) inhibitor, as well as immunotherapy drugs like the PD-1 inhibitor which is immune checkpoint inhibitor, are employed. With the continuous advancements in cancer clinical research, systematic strategies for cancer biomarkers, treatment, and prognosis have also rapidly developed, providing critical insights into the comprehensive clinical management of CCA. Biomarkers, known for their convenience, low cost, and dynamic monitoring capabilities, have garnered significant attention. Among these, exploring the potential role of DNA damage repair (DDR) alterations as predictive biomarkers in CCA has become a key research focus 5. Another prognostic scoring system, the Royal Marsden Hospital (RMH) Score, evaluates albumin levels, lactate dehydrogenase (LDH) levels, and the number of metastatic lesions through blood tests 6. Additionally, the neutrophil-to-eosinophil ratio (NER) has been identified as a prognostic indicator. Since neutrophils and eosinophils are critical components of the immune system, playing unique yet interrelated roles in cancer progression, changes in NER can also reflect the patient's prognosis 7. Immune checkpoint inhibitors (ICIs) have ushered in a new era of cancer treatment and have become a cornerstone for the management of advanced cancers. Despite their widespread use in the treatment of various cancers, including as neoadjuvant and adjuvant therapies, ICIs are associated with novel adverse events known as immune-related adverse events (irAEs). Studies have reported that most melanoma patients treated with ICIs experience hearing loss, which is correlated with a high response rate to ICIs 8. Furthermore, approximately 2-4% of cancer patients undergoing ICI therapy develop neurological irAEs, such as peripheral neuropathy and headaches 9. Thus, while ICIs demonstrate immense therapeutic potential in cancer management, careful consideration of irAEs and the development of effective management strategies are essential. Despite recent advancements in molecular medicine, pathological diagnosis, and treatment approaches for CCA, they have not kept pace with its rapidly increasing incidence and mortality rates. Due to tumor heterogeneity and subtle early symptoms, the diagnosis is often made at an advanced stage, and the existing treatment methods have shown limited efficacy, resulting in a poor prognosis for CCA 3. Therefore, efforts to improve early diagnosis, innovate treatment approaches, and identify effective prognostic biomarkers for CCA remain critical to enhancing patient survival and quality of life.

The 5-year survival rate for cholangiocarcinoma patients ranges from 7% to 20%, and the tumor recurrence rate after resection remains disappointingly high 10. Epidemiological investigations on the etiology of cholangiocarcinoma primarily differentiate between fluke-related and non-fluke-related CCA. In the Southeast Asian region, most cholangiocarcinoma cases are attributed to liver fluke infection. However, in other regions, several studies have indicated that primary sclerosing cholangitis 11, cirrhosis, non-alcoholic fatty liver disease (NAFLD), and hepatitis B have a stronger correlation with iCCA, while choledocholithiasis (bile duct stones) has a stronger relative with pCCA and/or dCCA 12, 13. While multiple risk factors contribute to the development of cholangiocarcinoma (CCA), no single, identifiable risk factor has been established. Pathological and molecular studies of CCA have revealed significant variations in etiology based on comprehensive genomic and epigenomic profiling. These studies indicate that different cancer subtypes within the same organ can arise through distinct extrinsic and intrinsic carcinogenic processes 14-16. Additionally, CCA tumors exhibit high levels of DNA methylation. Integrative analyses of somatic mutations and DNA methylation suggest that tumor initiation involves genetic driver mutations along with concurrent epigenetic alterations 12, 15. Metabolically, CCA undergoes significant shifts towards increased glycolysis and altered glycogen homeostasis to support rapid proliferation 17. Therefore, evaluating the interplay between genetic variations, metabolic changes, and CCA is critical for advancing cancer prediction and treatment strategies.

PGM2L1 is a member of the α-D-phosphohexomutase superfamily, which is found across all kingdoms of life 18. The enzymatic activity of recombinantly-expressed PGM2L1 was first characterized in 2007, identifying it as a glucose 1,6-bisphosphate (G16BP) synthase 19. PGM2L1 catalyzes the formation of G16BP by using the glycolytic intermediate 1,3-bisphosphoglycerate as a phosphate donor and glucose-1-phosphate or glucose-6-phosphate as a phosphate acceptor 20. Therefore, PGM2L1 plays a crucial role in glycogen metabolism, glycolysis, and gluconeogenesis as a key enzyme, and increasing evidence links its function to cancer metabolism and progression. Clinically, high PGM2L1 expression is associated with poorer prognosis in prostate and gastric cancers 21-23. However, despite clinical research data suggesting the potential involvement of PGM2L1 in cancer progression, the underlying molecular mechanisms of PGM2L1 in CCA development remain unclear.

In this study, we analyzed the correlation between PGM2L1 expression and clinical characteristics, as well as survival outcomes in CCA patients, using data from the TCGA database via UALCAN. Additionally, we investigated the methylation sites of PGM2L1 and its prognosis. We also explored its relationship with tumor-infiltrating immune cells in CCA. Finally, we performed functional enrichment analyses to compare the biological pathways associated with high and low PGM2L1 expression in CCA. To our knowledge, this study provides critical insights and brings clarity to the role of PGM2L1 in cholangiocarcinoma.

## 2. Materials and methods

### 2.1. PGM2L1 expression and overall survival analysis by UALCAN

Using an open website, UALCAN (https://ualcan.path.uab.edu/index.html) 24, to analyze the mRNA expression of PGM2L1 between Solid Tissue Normal patient samples and CCA samples. All the samples are from TCGA-CHOL (*n* = 45) database. Transcripts per million (TPM) expression values were calculated using an in-house PERL (Practical Extraction and Report Language) script. TPM was chosen as the measure of gene expression because it is considered to provide more accurate cross-sample comparisons than other metrics such as FPKM (Fragments Per Kilobase of transcript per Million mapped reads) and RPKM (Reads Per Kilobase of transcript per Million mapped reads). This dataset shows the gene-level transcription estimates, as in log2(x+1) transformed RSEM normalized count 25. The CCA samples were divided into two groups based on PGM2L1 expression: High expression (TPM values above the upper quartile) and Low expression (TPM values below the upper quartile) to plot a Kaplan-Meier survival curve of CCA patients (*n* = 9 in high expression and *n* = 36 in low expression).

### 2.2. Clinic data analysis and DNA methylation of the PGM2L1 gene

We extracted the clinic and DNA methylation data of the TCGA-CHOL project from UCSC Xena (http://xena.ucsc.edu). The correlations between prognosis and clinic-pathological parameters (Age, Gender, PGM2L1, Location, Stage) in CCA patients were analyzed. The DNA methylation data of TCGA-CHOL was measured using the Illumina Infinium HumanMethylation450 platform. The DNA methylation results of PGM2L1 and survival analysis were generated via R studio environment.

### 2.3. TIMER database to estimate tumor-infiltrating immune cells

The TIMER database (https://cistrome.shinyapps.io/timer/), which includes 10897. samples across 32 cancer types from TCGA, is a comprehensive and kindly resource for estimating the abundance of immune infiltrates cells, including B cells, CD4+ T cells, CD8+ T cells, neutrophils, macrophages, and DCs 26. We analyzed the correlation of PGM2L1 expression with the enrichment of immune infiltrates via the gene module. For each survival analysis, TIMER generates Kaplan-Meier plots for tumor-infiltrating immune cells (TIICs) and genes, showing survival differences between the upper and lower 50% of patients. The log-rank p-value is calculated and displayed on each plot.

### 2.4. Gene Ontology (GO), Kyoto Encyclopedia of Genes and Genomes (KEGG) and Gene Set Enrichment Analysis (GSEA)

We used computational methods, including GSEA 4.3.2 software and R Studio 4.4.1, to analyze the statistical significance of biological signaling pathways. In this study, GO and KEGG pathway analyses for PGM2L1-related genes were performed using the clusterProfiler package. GSEA classified genes based on their correlation with high- and low-PGM2L1 expression groups. Each analysis involved 1,000 gene set permutations. To rank enriched pathways for each phenotype, we utilized nominal p-values and normalized enrichment scores (NES). Pathways with a false discovery rate (FDR) < 0.05 were considered significantly enriched.

### 2.5. Statistical analysis

Multivariate Cox analysis was used to evaluate the influence of prognosis, PGM2L1. expression and other clinic-pathological factor. Additionally, some statistical analyses were accomplished with R software (packages: survminer, forestplot, pheatmap, ggplot2, limma, clusterProfiler, enrichplot). A two-tailed p<0.05 was considered to indicate statistical significance.

## 3. Results

### 3.1. High PGM2L1 expression and poor prognostic value in CCA

**Figure 1:**
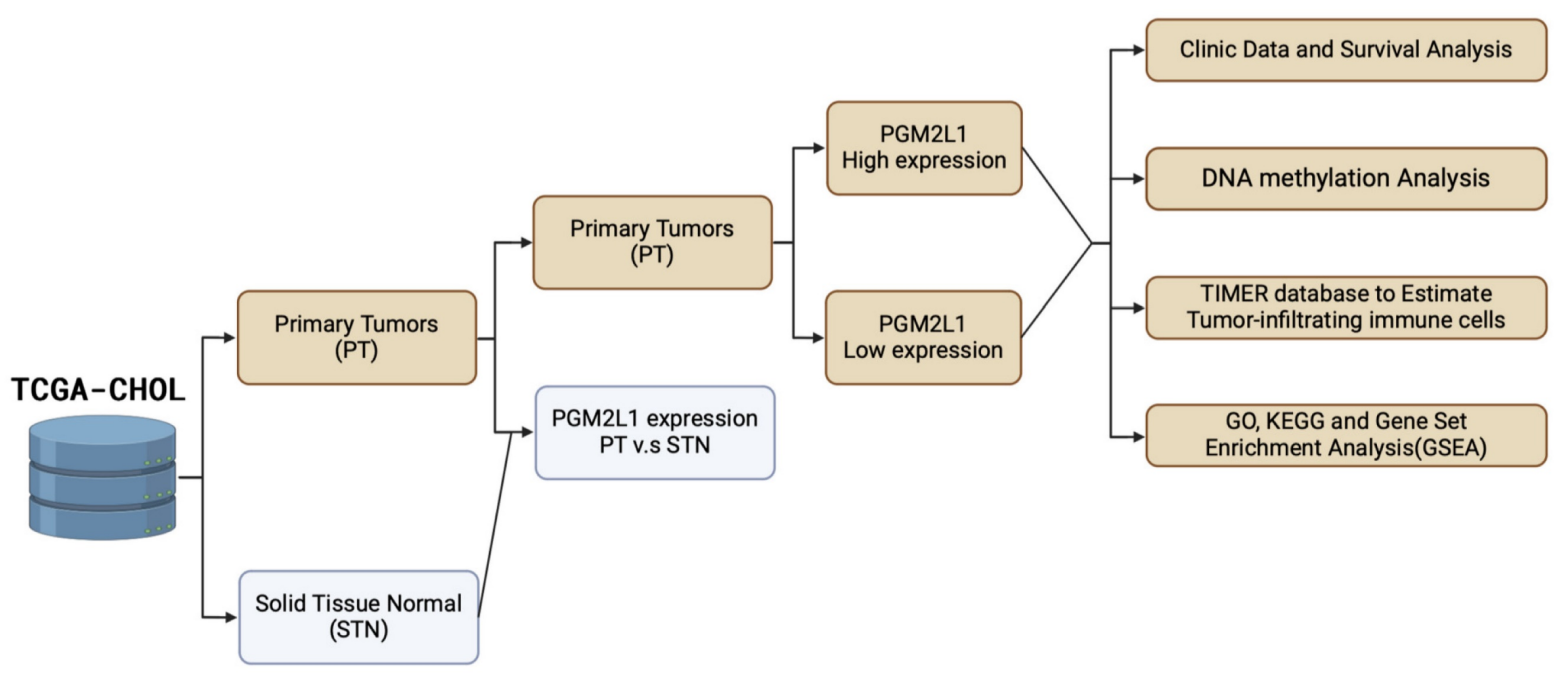
Workflow of the study analysis process. Workflow creation was performed using Biorender (https://app.biorender.com).

**Figure 2:**
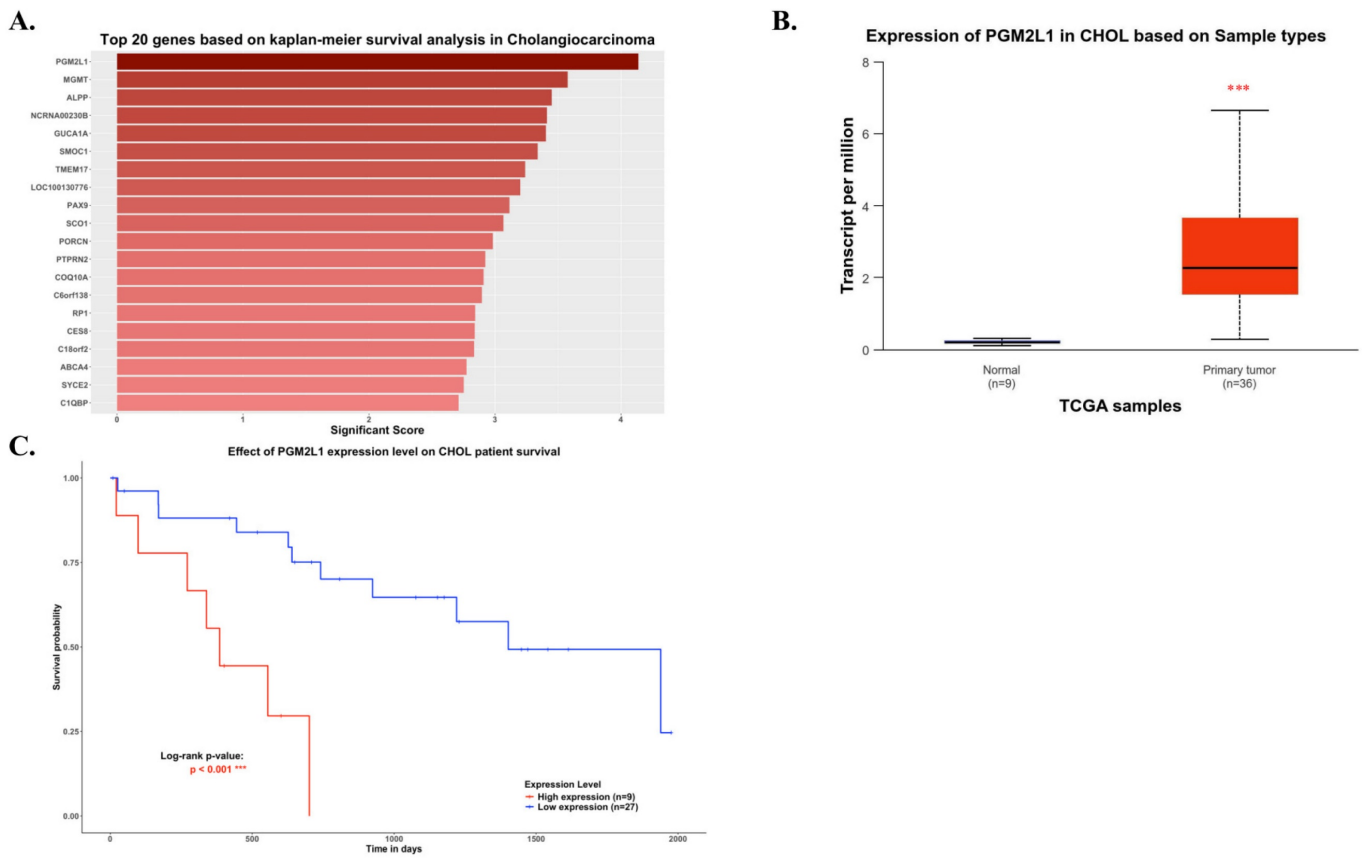
PGM2L1 ranked as the top gene in Kaplan-Meier analysis, showing differential mRNA levels between normal and CCA samples and correlating with overall survival in CCA. (A) Top 20 genes based on Kaplan-Meier survival analysis in TCGA-CHOL from UALCAN website (https://ualcan.path.uab.edu) (B) PGM2L1 mRNA expression is higher in normal tissue (n = 9) than that in CCA (n = 36) from the UALCAN website (https://ualcan.path.uab.edu). (C) Overall survival in CCA patients with high (red) and low (blue) PGM2L1 expression was analyzed using Kaplan-Meier survival curves. Mean ± SD, ***: p < 0.001.

As illustrated in Figure 1, the study was designed to assess the clinical significance of *PGM2L1* in CCA. Using data from the TCGA-CHOL project available on the UALCAN platform, we identified *PGM2L1* as a potential key prognostic gene through Kaplan-Meier survival analysis (Figure 2A). At the cellular level, CCA patients exhibited significantly higher mRNA expression of *PGM2L1* compared to normal people (Figure 2B). Furthermore, elevated PGM2L1 expression was associated with significantly worse prognosis in CCA patients (Figure 2C and Table 1). These findings suggest that *PGM2L1* may play a critical role in the pathogenesis of CCA through potential molecular interactions.

### 3.2. PGM2L1 methylation and survival in cholangiocarcinoma

**Figure 3:**
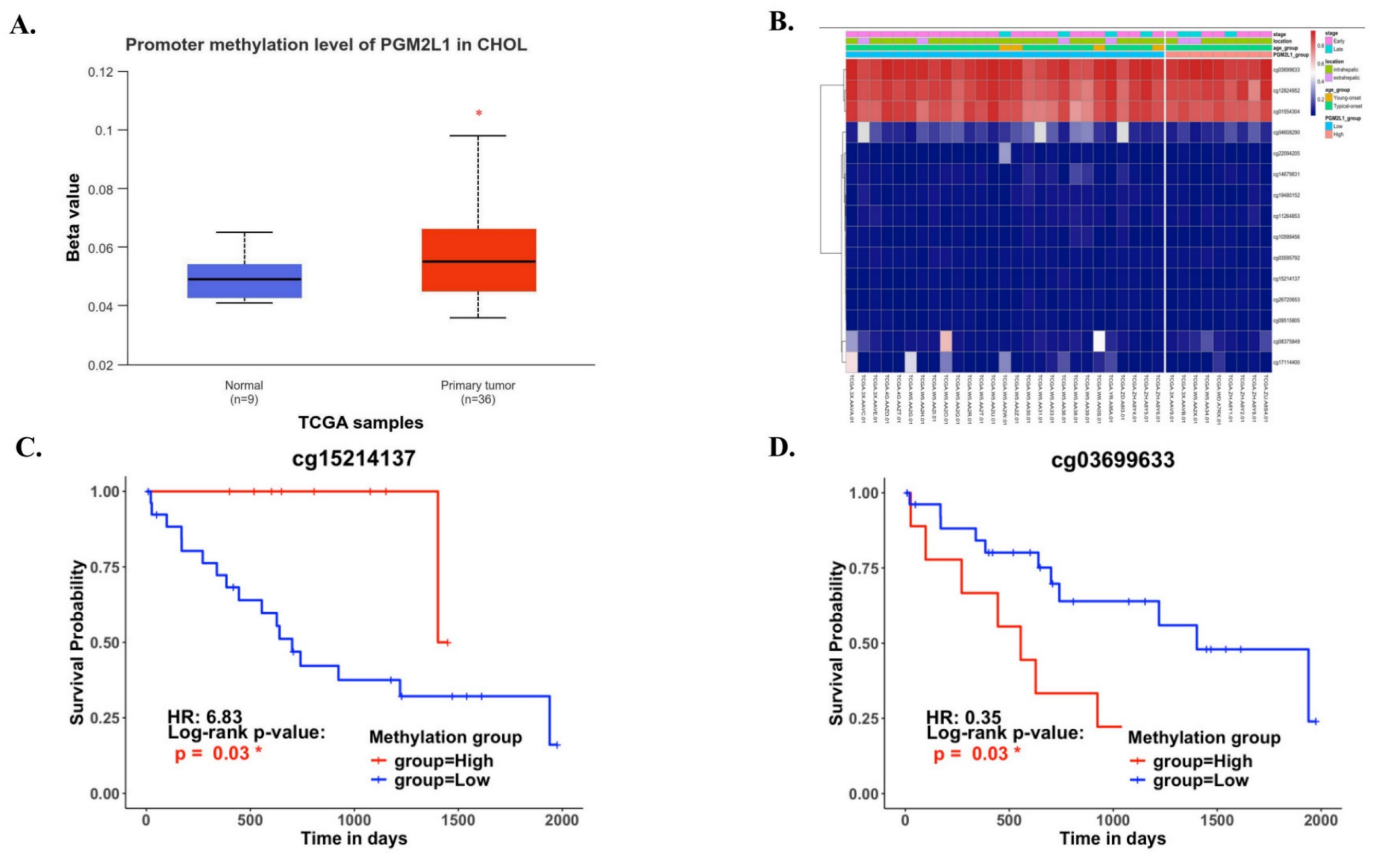
The DNA methylation of PGM2L1 in CCA of TCGA (A) Promoter methylation levels of PGM2L1 between Normal tissue and CCA. (B) Heatmap of DNA methylation expression level of PGM2L1 gene in CCA. (C-D) Prognostic value of single CpG of the PGM2L1 gene in CCA. The threshold of significance was Log-Rank Test p value <0.05.

DNA methylation is a crucial epigenetic modification involved in cancer progression. Methylation of CpG islands by DNA methyltransferases can act as a regulatory mechanism, either suppressing or promoting cell growth, and is reversible 27. In CCA patients, the promoter region of PGM2L1 exhibited significantly higher methylation levels compared to normal people (Figure 3A). A heatmap was generated to visualize the clustering of DNA methylation and expression levels of the PGM2L1 gene in CCA (Figure 3B). Moreover, specific DNA methylation sites, such as cg15214137 and cg03699633, were confirmed to be significantly altered (Figure 3C, and Table 2).

### 3.3. Correlation and prognostic of PGM2L1 and immune cell infiltration

**Figure 4:**
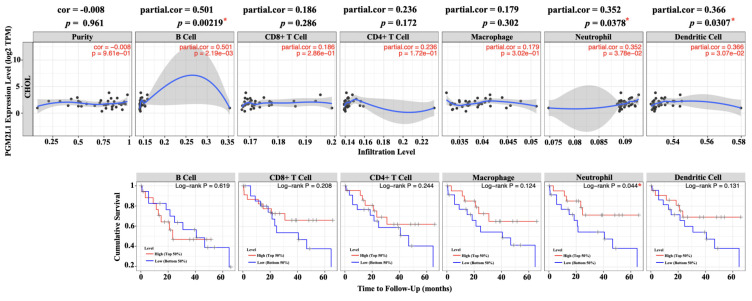
Correlation of PGM2L1 expression with immune infiltration level in CCA. (A) PGM2L1 expression levels in CCA from TCGA-CHOL database was determined by TIMER. (B) Prognostic roles of PGM2L1 and immune-related factor in CCA from TCGA-CHOL database was determined by TIMER. * p < 0.05.

Since the rise of immune checkpoint and immunotherapy concepts, increasing research has focused on the composition of immune cells within the tumor microenvironment. Understanding the interactions between tumors and immune cells, and identifying key immune factors, may pave the way for more effective cancer treatments 26. To this end, we utilized TIMER, a comprehensive web-based tool, to investigate the association between PGM2L1 expression and immune infiltration levels in cholangiocarcinoma (CCA). We implemented it by selecting PGM2L1 expression levels that were negatively correlated with tumor purity. The investigation showed that the level of PGM2L1 expression positively correlated with the infiltration level of B cell (*r* = 0.501, *p* = 0.00219), Neutrophils (*r* = 0.352, *p* = 0.0378) and DCs (*r* = 0.366, *p* = 0.0307) in CCA (Figure 4A). Moreover, our findings showed that Neutrophils (*p* = 0.044) is factor related to the cumulative survival rate of CCA over time (Figure 4B). These results strongly suggest that PGM2L1 is linked to immune infiltration in CCA.

### 3.4. Gene set and pathway enrichment analysis of PGM2L1

**Figure 5:**
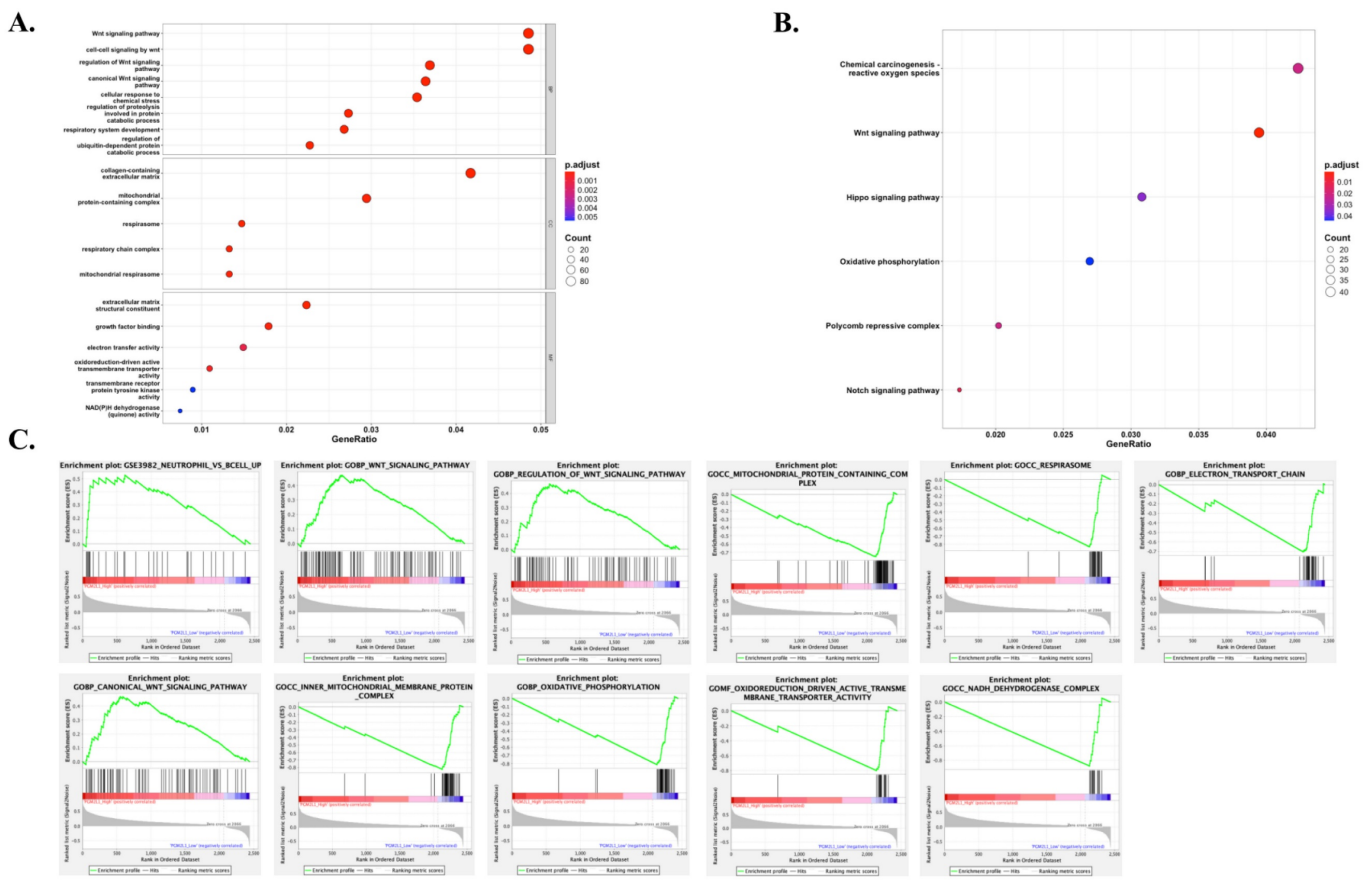
Gene sets and pathway enriched in PGM2L1 phenotype. (A) GO showed significant signaling pathways with PGM2L1 high and low expression groups. (B) KEGG showed significantly different signaling pathways with PGM2L1 high and low expression groups. (C) GSEA analysis revealed differential enrichment of genes with PGM2L1 high and low expression groups. (Nominal p-value < 0.05). GSEA, Gene Set Enrichment Analysis; GO, Gene Ontology; KEGG, Kyoto Encyclopedia of Genes and Genomes.

GSEA revealed significant differences in the enrichment of Gene Ontology (GO) terms and KEGG pathways between samples with high and low levels of PGM2L1 expression. We focused on listing the most significant GO terms and KEGG gene sets for both the high and low PGM2L1 expression groups (Figure 5). Notably, high PGM2L1 expression was associated with enrichment in the Wnt signaling pathway, glycolytic metabolism, and the attraction of neutrophils. These results suggest that PGM2L1 may play a role in regulating metabolic alterations and shaping the immune microenvironment.

## 4. Discussion

PGM2L1 was first identified as an enzyme involved in the synthesis of glucose-1,6-bisphosphate (G16BP) by Maliekal *et al.* in 2007. Increasing evidence has since supported the role of PGM2L1 in metabolic pathways, particularly glycolysis. In recent years, research on cancer metabolism has underscored the critical role of metabolic pathways in cancer development and therapy 28-30. Consequently, there has been increasing interest in glycolysis-related genes, including PGM2L1, in relation to cancer progression. Although PGM2L1 is expressed in various tumors, including brain, prostate, and gastric cancers, its role in tumor development appears to vary across different cancer types 20-22, 31. However, the precise mechanisms by which PGM2L1 regulates tumor progression and its potential prognostic value, particularly in CCA, remain unclear.

In this investigation, we first dissected PGM2L1 expression in CCA patients and found that PGM2L1 may play a central role in CCA progression. DNA methylation, which is crucial for tumorigenesis and cancer progression, is particularly significant in CCA 32. Due to its genetic heterogeneity, CCA presents unique therapeutic challenges, prompting substantial interest in the clinical applications of cancer DNA methylation, especially in identifying prognostic markers 33. Therefore, we identified the methylation site of PGM2L1 in CCA and its prognostic significance, indicating that CCA may utilize distinct methylation sites to modulate tumor development.

The tumor microenvironment (TME) refers to the complex ecosystem surrounding a tumor, consisting of various cell types, including immune cells, fibroblasts, and endothelial cells, as well as the extracellular matrix, blood vessels, and soluble factors. The TME plays a pivotal role in cancer development and has been suggested to influence the response to treatments. Given these considerations, there is increasing interest in the TME as a target for developing effective anticancer therapies 34. TIMER analysis revealed that high PGM2L1 expression is positively correlated with immune infiltration levels, particularly in B cells, neutrophils, and dendritic cells, with neutrophils identified as significant independent risk factors in CCA. Whether the effect of PGM2L1 on the prognosis of CCA patients is closely linked to neutrophils remains to be further investigated. Notably, there are currently no relevant reports addressing this association.

To further investigate the function of PGM2L1 in CCA, we performed GSEA using TCGA dataset. GSEA showed that signaling pathways, mainly associated with neutrophils infiltration and Wnt signaling pathway were significantly enriched in PTPRN high expression, in contrast, oxidative phosphorylation and mitochondrial respiration were significantly enriched in PGM2L1 low expression. Extensive research has linked the Wnt signaling pathway and metabolic processes, particularly glycolysis, to cancer progression. Notably, Wnt-driven Warburg metabolism directs glucose utilization toward cancer cell proliferation and enhances the delivery of oxygen and nutrients via blood vessels. Additionally, it plays a critical role in promoting proliferation, invasion, apoptosis, and angiogenesis, while driving both glycolytic and energetic metabolism 35-38. Therefore, PGM2L1 may be involved in regulating these pathways, potentially altering the progression of CCA.

This study holds significant potential in advancing our understanding of CCA by elucidating the role of PGM2L1 in tumor progression and immune modulation. By identifying PGM2L1 as a prognostic marker, it opens avenues for targeted therapies aimed at metabolic pathways, potentially improving patient outcomes. However, knowledge gaps persist, particularly regarding the precise mechanisms by which PGM2L1 influences the tumor microenvironment and its interaction with immune cells. To address these gaps, we will employ integrative trans-omics approaches, combining genomic, transcriptomic, and proteomic analyses, alongside cellular experiments and clinical validation. Such comprehensive strategies have been effective in identifying diagnostic biomarkers and therapeutic targets in other cancers 22. In the next five years, we are likely to witness a deeper exploration into the metabolic reprogramming of cancer cells and its impact on immune evasion. Studies focusing on the immunological aspects of cancer cell metabolism will be crucial in developing novel therapeutic interventions that can disrupt the metabolic pathways facilitating tumor growth and immune suppression 39. Unfortunately, the limitation of this study is the reliance on *in vitro* models and established CCA cell lines, which may not fully recapitulate the complex heterogeneity and microenvironment of human tumors. While these models provide critical mechanistic insights, they cannot entirely mimic the dynamic interactions within the TME. To address this limitation, future work will incorporate patient-derived organoids and xenograft models to validate key findings in systems that better represent the clinical landscape.

## 5. Conclusions

Our study demonstrates that PGM2L1 expression is linked to the recruitment of immune cells, particularly neutrophils. Additionally, PGM2L1 appears to regulate key pathways in CCA, including neutrophil infiltration, tumor metabolism, and the Wnt signaling pathway, all of which may contribute to tumor progression. To our knowledge, this is the first study to reveal a strong interaction between PGM2L1 expression and CCA. These results suggest that PGM2L1 may serve as an independent prognostic factor and is closely linked to the tumor microenvironment in CCA.

## Figures and Tables

**Table 1:**
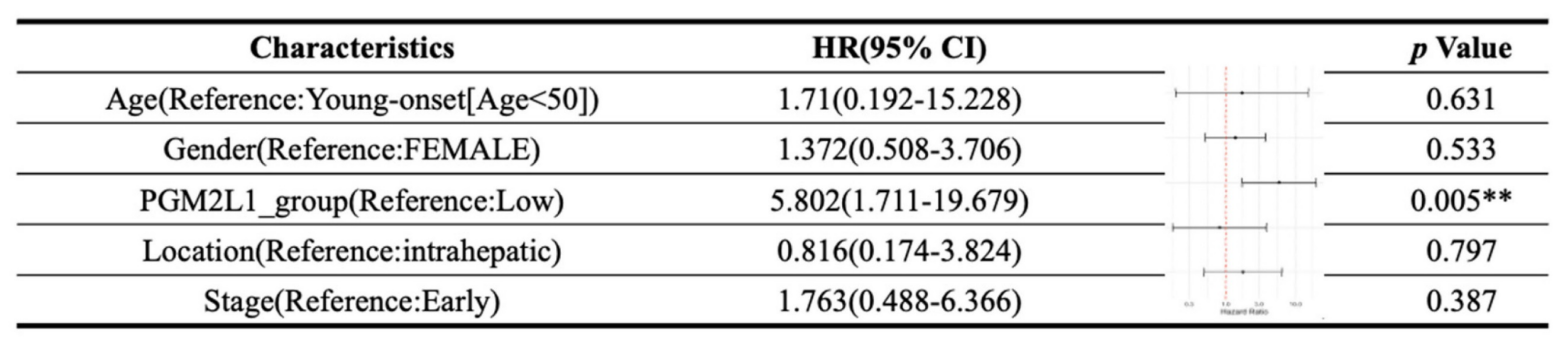
Multivariate Cox regression analysis of clinicopathological features (including PGM2L1 expression) with OS in the TCGA datasets.

**p value <0.01 as statistically significant

**Table 2:**
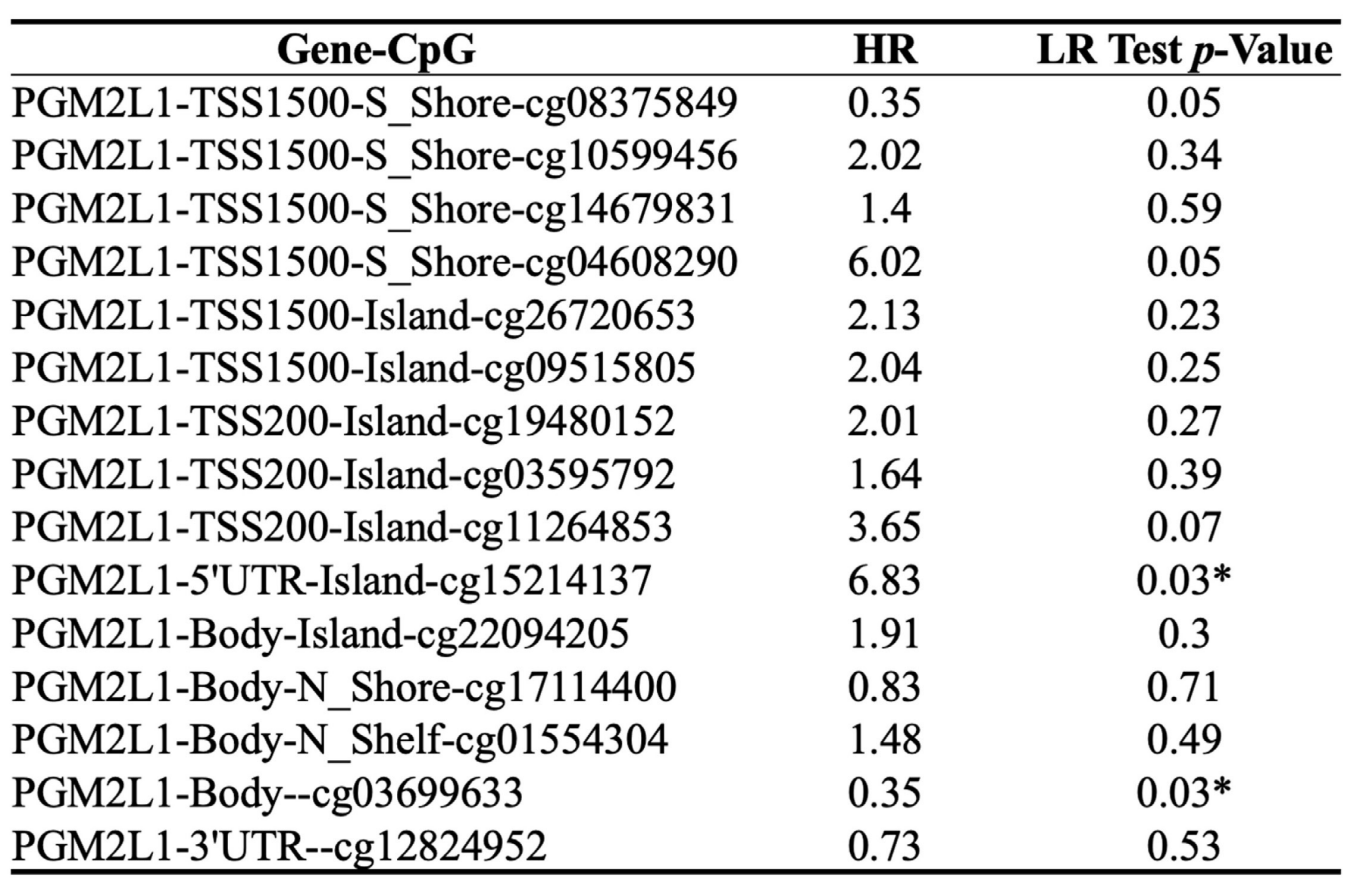
Prognostic Value of Single CpG of the PGM2L1 gene family in CCA by HumanMethylation450 platform.

* *p* value < 0.05 as statistically significant
